# Weight Loss Improves HRQOL Physical Function and Vitality More in Blacks Than Whites

**DOI:** 10.1093/geroni/igaa057.3079

**Published:** 2020-12-16

**Authors:** Robert Boudreau, Elizabeth Venditti, Michelle Danielson, Nancy Glynn, John Jakicic, Anne Newman, Steven Albert

**Affiliations:** University of Pittsburgh, Pittsburgh, Pennsylvania, United States

## Abstract

Participant-reported outcomes are important. Prior MOVE UP reports show ≥5% weight loss was not significantly associated with depressive symptoms but was associated with positive SPPB physical function and the Physical Component Score of the SF-36 HRQOL scale. We examined the SF-36 subscales that showed, a priori, clinically meaningful +5.0-point increases over 13 months, the change in subscales per 5% weight loss, and variability by race. Among all participants (n =240) several subscales show significant pre-post changes [mean (SD)] but only Vitality [+5.6 (15.4)] and Physical Function [+5.0 (16.7)] meet the criterion. Blacks (n = 60) compared to Whites (n = 172) had higher baseline scores on these subscales, were less likely to lose ≥5% (31.7% vs. 59.9%), but mixed regression models indicate that those who did demonstrated a larger change on Vitality (+5.2; p<0.048) than Whites (+3.1; p<0.0003). Studying weight loss and HRQOL associations in larger minority samples is needed.

